# Assessment of General Populations Knowledge, Attitude, and Perceptions Toward the Coronavirus Disease (COVID-19): A Cross-Sectional Study From Pakistan

**DOI:** 10.3389/fmed.2021.747819

**Published:** 2021-12-24

**Authors:** Saadullah Khattak, Maqbool Khan, Tahir Usman, Johar Ali, Dong-Xing Wu, Muhammad Jahangir, Kashif Haleem, Pir Muhammad, Mohd Ahmar Rauf, Kamran Saddique, Nazeer Hussain Khan, Tao Li, Dong-Dong Wu, Xin-Ying Ji

**Affiliations:** ^1^Henan International Joint Laboratory for Nuclear Protein Regulation, School of Basic Medical Sciences, Henan University, Kaifeng, China; ^2^Sino-Pak Center for Artificial Intelligence, Pak-Austria Fachhochschule, Institute of Applied Sciences and Technology, Haripur, Pakistan; ^3^College of Veterinary Science and Animal Husbandry, Abdul Wali Khan University Mardan, Mardan, Pakistan; ^4^Vice-Chancellor in Khushal Khan Khattak University Karak, Karak, Pakistan; ^5^State Key Laboratory of Earth Surface Processes and Resource Ecology, Faculty of Geographical Science, Beijing Normal University, Beijing, China; ^6^Department of Psychiatric and Mental Health, Central South University, Changsha, China; ^7^Department of Microbiology, Hazara University, Mansehra, Pakistan; ^8^Henan-Macquarie University Joint Centre for Biomedical Innovation, School of Life Sciences, Henan University, Kaifeng, China; ^9^Department of Surgery, Miller School of Medicine, University of Miami, Miami, FL, United States; ^10^College of Communication, East China Normal University, Shanghai, China; ^11^School of Stomatology, Henan University, Kaifeng, China; ^12^Kaifeng Key Laboratory of Infection and Biological Safety, School of Basic Medical Sciences, Henan University, Kaifeng, China

**Keywords:** knowledge, attitude, perceptions, coronavirus disease 2019, Pakistan

## Abstract

**Background:** Coronavirus disease 2019 (COVID-19) is a global health threat and caused a universal psychosocial impact on the general population. Therefore, the knowledge, attitude, and perceptions (KAPs) of the general population are critical for the development and effective implementation of standard operating procedures (SOP) to contain the contagion and minimize the losses. Therefore, the current study was conducted to understand and evaluate the KAPs of Pakistani populations toward the COVID-19.

**Methods:** An online cross-sectional study was carried out among participants from 1 May to 30 July 2020 in different areas of Pakistan. The respondents of the study were the general population with age ≥ 18 years. The poll URL was posted on several channels after a call for participation. Other social media platforms such as WeChat, WhatsApp, Facebook, Twitter, Instagram, Messenger, and LinkedIn were engaged to maximize general population engagement. The questionnaire included details about sociodemographic, knowledge about COVID-19, perceptions toward universal safety precautions of COVID-19, and beliefs attitude toward the COVID-19. The obtained data were exported into a Microsoft Excel spreadsheet and SPSS software version 21 for windows. The descriptive statistics values were presented in frequencies and percentages. Binary logistic regression, Chi-square test, and one-way ANOVA were applied to analyze the participants' socio-demographic characteristics and variables related to KAPs. *P-value* < 0.05 was recorded as significant.

**Results:** A total of 1,000 participants were invited of which 734 participated in this study. The response rate was 73.4% (734/1,000). The gender, marital status, education, and residence showed a significant association with the knowledge score. The majority of the study participants were thinking that COVID-19 may be more dangerous in elderly individuals 94.5% (*n* = 700), and individuals with chronic diseases or severe complications 96.7% (*n* = 710) (*p* = 0.00). More than half of the participants 52.5% (*n* = 385) showed their concern that either they or their family members might get the infection. More than 98% (*n* = 703), (P-value = 0.00) of the participants held that COVID-19 would be successfully controlled in Pakistan by following the standard SOPs and government guidelines.

**Conclusion:** This study showed that the general population of Pakistan has good awareness and reasonable attitudes and perceptions toward the full features of the COVID-19. The current study suggests that mass-level effective health education programs are necessary for developing countries to improve and limit the gap between KAP toward COVID-19.

## Introduction

Coronavirus disease 2019 (COVID-19) was first noticed in Wuhan city of Hubei Province, China in late December 2019 (1). On March 11, 2020, the World Health Organization (WHO) declared it a pandemic (2, 3). The pandemic paralyzed the healthcare system in many countries due to its rapid spread (4). On July 11, 2021, the disease infected ~190 million people and caused more than four million deaths across the globe (5). Both the developed and developing parts of the world were hit hard by the pandemic and resultantly huge mortalities were reported in the United States of America (USA), India, and Brazil (6, 7). The overall number of confirmed cases in low-resource or developing nations is projected to rise dramatically shortly. COVID-19 was discovered to be a growing threat to the global economy, particularly China's surrounding developing nations, including Pakistan, India, Iran, Afghanistan, and Bangladesh (8). It is reported that developed countries have defined health care systems and public education programs as compared to low- and middle-income countries (LMICs). It is crucial for LMICs, including Pakistan, to strengthen their healthcare systems to save lives, ensure patients' quality of life, and assure patient safety and well-being of the general population (9).

Pakistan is a densely populated country with around 197 million people having a low to the medium-level economy. It comprises four provinces i.e., Punjab, Sindh, Khyber Pakhtunkhwa (KP), Balochistan, and three territories that include Islamabad Capital Territory, Gilgit–Baltistan, Azad Jammu, and Kashmir (10). The bell for the approaching storm was rung out on 26 February 2020, when the first two cases of COVID-19 were reported in Pakistan (11). There were 973,284 confirmed COVID-19 cases and 22,582 deaths across the country when writing this article (12, 13). The COVID-19 outbreak harshly hit Punjab province as it has the highest number of COVID-19 cases (with 348,000 cases as of 11 July 2021) (12, 13).

The government of Pakistan devised strategies such as partial lockdown, social distancing, closure of schools, colleges, and universities across the country, travel restrictions, suspension of general populations transportation, quarantine centers, isolation wards, and diagnostics laboratories, among others, to deal with the pandemic and contain the contagion. Despite these controls, COVID-19 instances continued to be reported regularly. These control mechanisms are said to work because of the general population's knowledge, behavior, and compliance. With the massive distribution of misinformation and falsehoods about COVID-19 on various online networking and social media platforms, the pandemic became more infodemic, making it difficult for the general population to know which sources to believe. To forestall individuals' misconception about this viral malady, the WHO needed to dispatch a page entitled “Myth busters” on their website page (14). The adequacy of government-run data campaigns fundamentally relies upon what individuals see, anticipate, and think about COVID-19. Therefore, it is critical to teach the general population in general about cleanliness standards, the spread of sicknesses, and potential alternatives to treat them; thus making the control measures work well. Following other countries, The government of Pakistan must analyze the general population's knowledge, attitude, and practice or perceptions (KAP) and then develop strategies and standard operating procedures (SOPs) to effectively combat the epidemic (15, 16). Therefore, an online survey was performed to determine KAPs of the Pakistani population toward COVID-19.

## Methods

### Study Design and Procedure

Online/web-based study was designed from 1 May to 30 July 2020. The subject population of the study was the general population with only one restriction of age i.e., ≥ for 18 years, except age; there were no other exclusion criteria. a total of 1,000 individuals were invited. The response rate was 73.4% (734/1,000). According to the World Population Review, Pakistan's reported population on July 1, 2021, was 225,199,937 (17). The Raosoft sample size calculator was used for the required sample. The calculated minimum required sample size was 385 (18). For sample estimation, the response distribution was assumed to be 50%, the margin of error was set at 5%, and the confidence level was set at 95%. The poll URL was posted on several channels after a call for participation. Other social media platforms such as WeChat, WhatsApp, Facebook, Twitter, Instagram, Messenger, and LinkedIn were engaged to maximize general population engagement. It took about 10 min to complete the survey. Only the core members had access to the repository data, which ensured privacy.

### Questionnaire Instruments and Variables

A structured questionnaire was established by reviewing the relevant available scientific literature and published resources on COVID-19 (1). The questionnaire was written in the English language, which is the primary language of higher education and the official language of Pakistan. Four different sections were developed in the used questionnaire: (1) socio-demographic information, (2) knowledge about COVID-19, (3) perceptions toward universal safety precautions of COVID-19, and (4) attitude of study participants toward the COVID-19.

### Socio-Demographic Information

The socio-demographic characteristics section collected information on the participant's gender, age, residential area, level of education, marital status, employment status, and native language. The participants' education levels were classified as uneducated, school, college, graduate, and post-graduate. Similarly, the participant's occupation was classified as full-time, part-time, seeking opportunities, retention, student, housewife, scholarship student, and so on, and the participant's native language response included Punjabi, Pashto, Sindhi, and Saraiki, Balochi, Urdu, Shina, and Hindko languages.

### COVID-19 Related Knowledge

The questions included in this section are related to COVID-19 symptoms such as fever, dry cough, body pain, sour throat, chest pain or pressure, and loss of taste or smell. Questions like antibiotics use against the COVID-19; an effective treatment against the virus is currently available; the virus may be more dangerous for the elderly individuals, the virus may be more dangerous in patients with chronic diseases, and different aspects of preventive measures such as the concept of proper hand wash and avoiding handshaking, wearing facemasks in general populations places and not sharing personal items, avoiding crowds, eating or drinking in general populations places during the COVID-19 outbreak, were included in the survey.

### Perceptions Related to Universal Safety Precaution of COVID-19

Two questions were asked to assess the participant's perceptions of the COVID-19 universal safety precaution. It was scored on a Likert–type scale of Yes, No, and Not sure. For example, I am concerned about the possibility that another family member or I may become infected with this virus, and I believe the media coverage of this disease is exaggerated.

### Attitude

Three questions were asked to assess the general population's attitude toward COVID-19 including the general population's confidence in the government to win the fight against COVID-19, the government's implementation of curfews, and lockdowns, and general populations support for strict policies if they help protect the vulnerable.

### Statistical Analysis

For data analysis, the participants' responses in Google form were transferred to Microsoft Excel and then imported into IBM SPSS software version 21.0 for Windows (IBM Corp. Armonk, NY, USA). Participants' demographic characteristics were described by descriptive statistics such as frequency, percentage, mean and standard deviation (SD). For categorical data, numbers and percentages were provided, whereas mean and SD were given for continuous data. The independent *t*-test, one-way ANOVA, and (Chi-square test and binary logistic regression) analysis of variance were employed to evaluate the significance levels. A *P* = 0.05 was declared statistically significant.

### Ethical Consideration

The current study was approved by the Ethical Research Committee Department of Microbiology, Hazara University, Pakistan (study registration No: Micro/BC/2021/15). Our web-based survey first page had an informed consent statement, and it required participants to consent (accept or decline) to participate. This was a voluntary survey, and participants were provided with no incentive.

## Results

### General Characteristics

A total of 734 study participants were recruited in the final analysis, of which 80.7% were males and only 19.20 were females as shown in Table 1. About two-thirds of the participants (65.25%) aged 18– <30 years, one-quarter of participants (22.47%) aged between 30 and 40 years, 40–50 years aged participants were 8.03%, 50–60 aged were 2.3% and 60–70 years of aged participants contributed only 1.9%. The majority of the study participants were from rural areas (56.5%), followed by urban areas (41.1%), and other areas (2.2%). The level of knowledge includes graduates up to 38.0 %, postgraduate 14.2 %, college-level 33.8 %, and school education 8.7 %. Only 5.2 % were uneducated. The marital status results show that single participants were 56.9 and 42.9% were married. According to our survey, 40.2% were Pashto speakers, 11.7% were Punjabi speakers, and 40.2% were Urdu speakers.

**Table 1:** Demographic characteristics of participants (*N* = 734).

**Characteristics**		**Frequency**	**Percentage(%)**	**Valid percentage**	**Cumulative percentage**
Gender	Male	593	80.70	80.80	80.80
	Female	141	19.20	19.20	100.0
Age	18 <30	479	65.25	65.26	65.26
	30 <40	165	22.47	22.48	87.74
	40 <50	59	8.03	8.04	95.78
	50 <60	17	2.3	2.32	98.10
	60 <70	14	1.9	1.90	100.0
Area of Residence	Others	16	2.2	2.2	2.3
	Rural	415	56.5	56.5	58.9
	urban	302	41.1	41.1	100
Education	Collage	248	33.8	33.8	33.9
	Graduate	279	38.0	38.0	71.9
	Postgraduates	104	14.2	14.2	86.1
	School	64	8.7	8.7	94.8
	Uneducated	38	5.2	5.2	100.0
Marital Status	Married	315	42.9	42.9	43.1
	Single	418	56.9	56.9	100.0
Employment status	Full-Time	291	39.6	39.6	39.8
	Housewife	3	0.4	0.4	40.2
	Part-Time	337	45.9	45.9	86.1
	Retired	13	1.8	1.8	87.9
	Seeking opportunities	51	6.9	6.9	94.8
	Student	16	2.2	2.2	97.0
	Unemployed	22	3.0	3.0	100.0
Native Language	Balochi	7	0.95	0.95	0.95
	Hindko	2	0.27	0.27	1.22
	Pashto	295	40.2	40.19	41.41
	Punjabi	86	11.7	11.72	53.13
	Saraiki	23	3.1	3.14	56.27
	Shina	2	0.27	0.27	56.54
	Sindhi	24	3.3	3.27	59.81
	Urdu	295	40.2	40.19	100.0

### COVID-19 Related Knowledge

In total, 44.3% of participants thought that antibiotics could treat the COVID-19 disease, 95.4% of participants' views that the virus is riskier for elder people. 96.7% of participants said that the virus is more hazardous in patients with chronic diseases, 45.6% response that an effective treatment against the virus is currently available.

99.3% of participants thought of Fever, dry cough, body aches, and sore throat, 90.5% participants thought that loss of taste or smell, and 84.2% participants thought chest pain or pressure was a common symptom of COVID-19.

**Figure 1:**
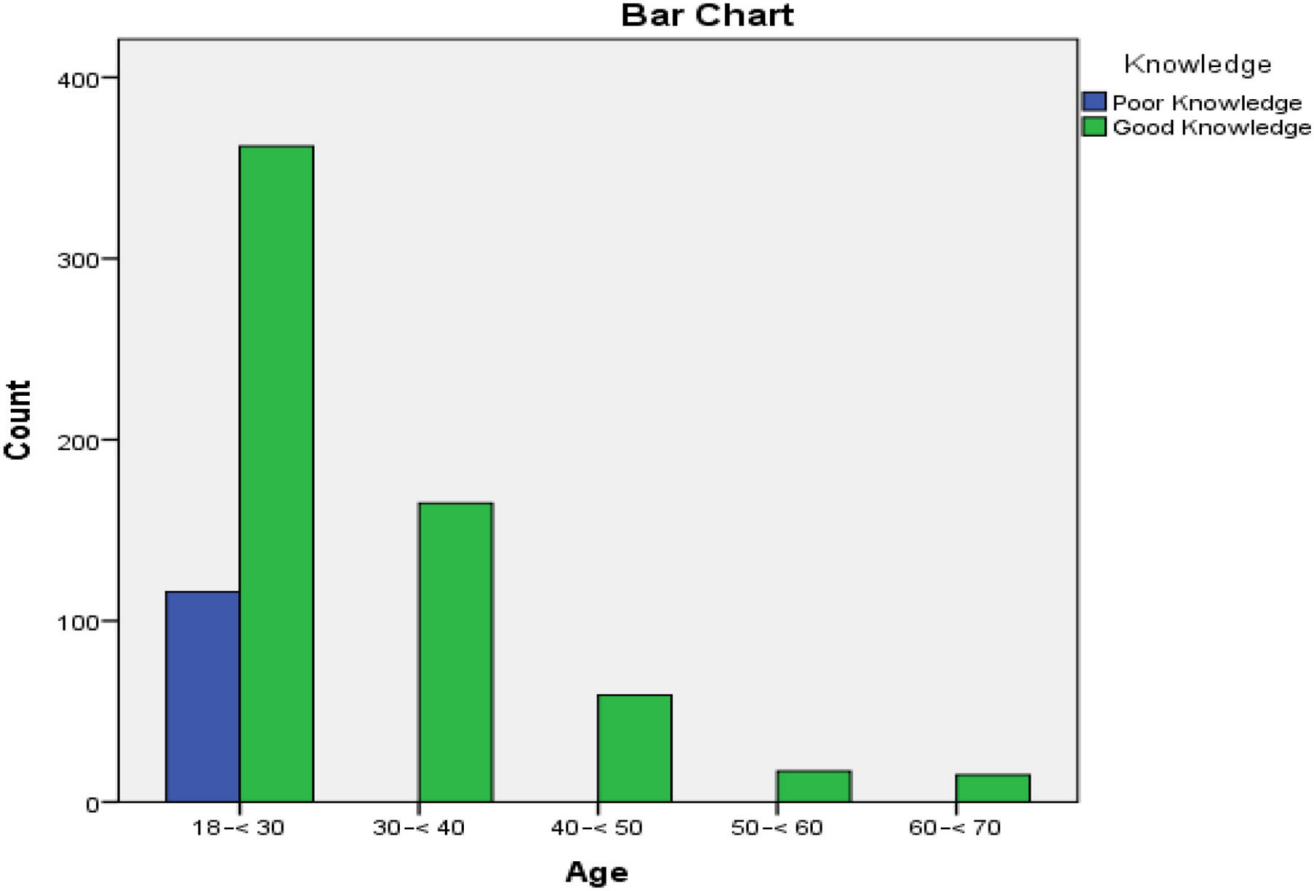
Age-based knowledge response of the study participants toward COVID-19.

In the item of measures to prevent the spread of the disease, 99.9% of participant's thought proper handwashing and avoid handshaking, wearing facemasks in general populations places and not sharing personal items (99.0%), avoiding crowds, eating, or drinking in general populations places (98.00%) as a measure to prevent the spread of the disease as shown in Figure 1.

Results of knowledge assessment of the participants regarding spread, common symptoms, and measures to prevent the spread of COVID-19 are shown in Tables 2, 3 and Supplementary Tables 1, 2.

**Table 2:** Participant knowledge of COVID-19 (*N* = 734).

**Questions**	**Responses**	**Frequency**	**Percent**	**Valid percent**	**Cumulative percent**
Antibiotics are an effective treatment for COVID-19	Maybe	106	14.4	14.4	14.4
	No	303	41.3	41.3	55.7
	Yes	325	44.3	44.3	100.0
An effective treatment against the virus is currently available	Maybe	48	6.5	6.5	6.5
	No	351	47.8	47.8	54.4
	Yes	335	45.6	45.6	100.0
The virus may be more dangerous for the elderly individuals	No	17	2.3	2.3	2.3
	Not sure	17	2.3	2.3	4.6
	Yes	700	95.4	95.4	100.0
The virus may be more dangerous in patients with chronic diseases	No	7	1.0	1.0	1.0
	Not sure	17	2.3	2.3	3.3
	Yes	710	96.7	96.7	100.0
**Common symptoms include**,	No	4	0.5	0.5	0.5
i) Fever, Dry Cough, Body Pain, Sour Throat.	Not Sure	1	0.1	0.1	0.7
	Yes	729	99.3	99.3	100.0
ii) Chest pain or pressure	No	88	12.0	12.0	12.0
	Not sure	28	3.8	3.8	15.8
	Yes	618	84.2	84.2	100.0
iii) Loss of taste or smell	No	37	5.0	5.0	5.0
	Not sure	33	4.5	4.5	9.5
	Yes	664	90.5	90.5	100.0
**Measure to prevent the spread of the disease include**,	No	1	0.1	0.1	0.1
i) Proper handwashing and stop shaking hands	Yes	733	99.9	99.9	100.0
	No	4	0.5	0.5	0.5
ii) Putting on facemask in public places and do not share personal items	Not sure	3	o.4	0.4	1.0
	Yes	727	99.0	99.0	100.0
iii) Do not gather in groups and avoid eating or drinking in a public place	No	11	1.5	1.5	1.5
	Not sure	4	0.5	0.5	2.0
	Yes	719	98.0	98.0	

**Table 3:** Association of knowledge with demographic variables of respondents (*N* = 734).

**Variables**	**Categories**	**Knowledge score (Mean+-SD)**	**t/F**	**p-value**
Gender	Male	8.18 ± 1.686	125.11	0.00 (<0.05)
	Female	10.00 ± 0.00		
Age	18 <30	7.74 ± 1.593	127.703	0.00 (<0.05)
	30 <40	10.00 ± 0.00		
	40 <50	10.00 ± 0.00		
	50 <60	10.00 ± 0.00		
	60 <70	10.00 ± 0.00		
Area of Residence	Urban	10.00 ± 0.000	1178.801	0.00 (<0.05)
	Rural	7.70 ± 1.063		
	Others	2.25 ± 1.125		
	Collage	6.90 ± 1.632		0.00 (<0.05)
Education	Graduate	8.88 ± 0.977	224.046	
	Postgraduates	10.00 ± 0.000		
	School	10.00 ± 0.000		
	Uneducated	10.00 ± 0.000		
Marital Status	Married	7.14 ± 1.515	25.925	0.00 (<0.05)
	Single	9.57 ± 0.805		
Employment Status	Full-Time	7.07 ± 1.556	109.291	0.00 (<0.05)
	Housewife	8.00 ± 0.000		
	Part-Time	8.00 ± 0.901		
	Retired	10.00 ± 0.000		
	Seeking opportunities	10.00 ± 0.000		
	Student	9.65 ± 0.786		
	Unemployed	9.43 ± 0.926		
Native Language	Balochi	1.14 ± 0.690	388.265	0.00 (<0.05)
	Hindko	3.00 ± 0.000		
	Pashto	7.27 ± 1.193		
	Punjabi	8.00 ± 0.000		
	Saraiki	8.78 ± 0.736		
	Shina	10.00 ± 0.000		
	Sindhi	10.00 ± 0.000		
	Urdu	10.00 ± 0.000		

### Perceptions of the Study Participants

**Figure 2:**
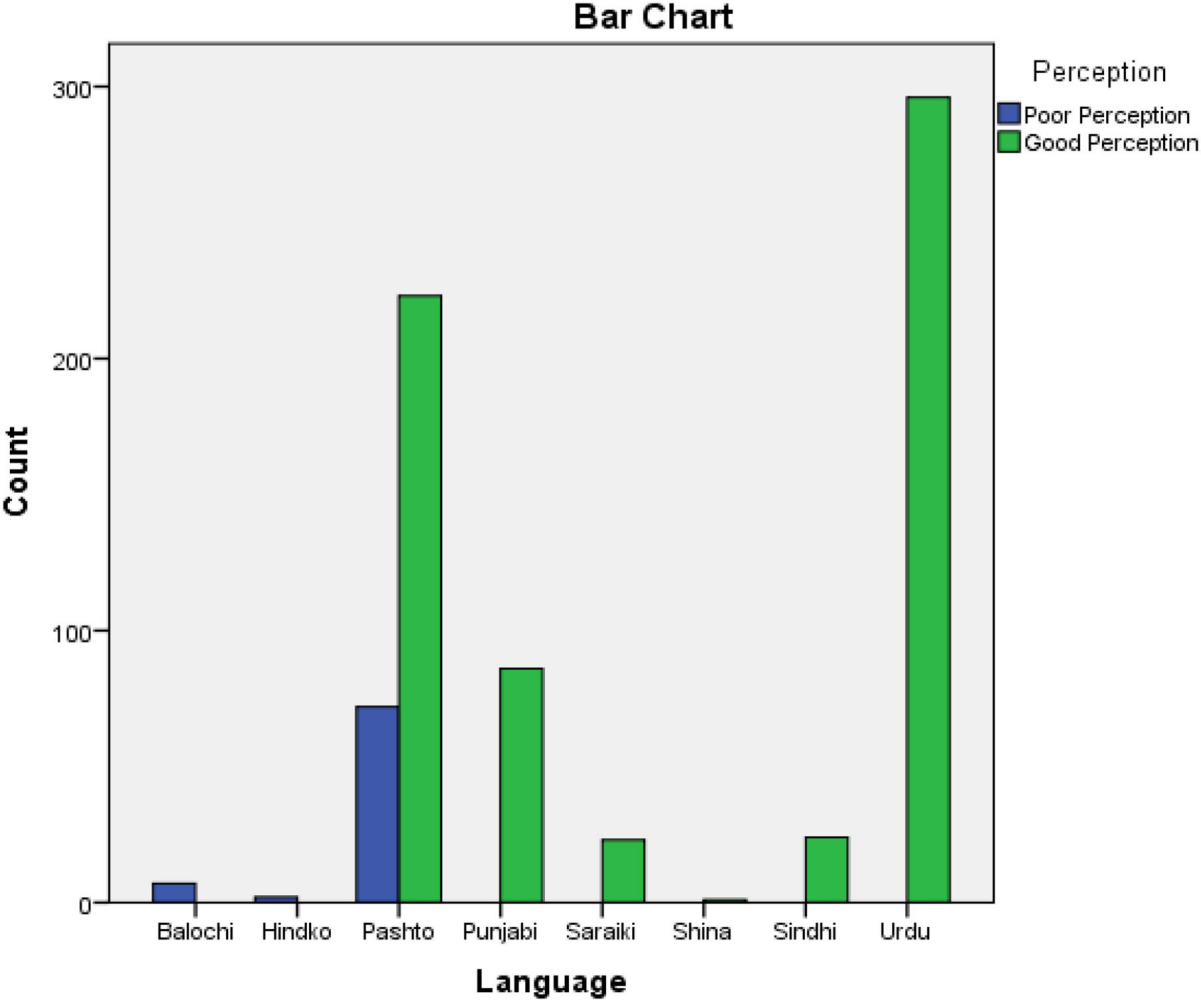
Language-based response of participants to perception.

Our results show that 52.5% of participants were worried about the probability that another family member or I can get infected with this virus, 89.0% of participants consider the media coverage about this disease is overstated; Tables 4, 5 and Supplementary Tables 1, 3 provides the details of these items and see Figure 2.

**Table 4:** Perception toward COVID-19 (*N* = 734).

**Questions**	**Responses**	**Frequency**	**Percent**	**Valid percent**	**Cumulative percent**
**Perception toward COVID-19**,					
i) I am concerned about the possibility that another family member or I can get infected with this virus	No	313	42.6	42.6	42.6
	Not sure	36	4.9	4.9	47.5
	Yes	385	52.5	52.5	100.0
ii) I think media courage about this disease is exaggerated	No	34	4.6	4.6	4.6
	Not sure	47	6.4	6.4	11.0
	Yes	653	89.0	89.0	100.0

**Table 5:** Association of perception with demographic variables of respondents (*N* = 734).

**Variables**	**Categories**	**Perception score (Mean+-SD)**	**t/F**	**p-value**
Gender	Male	1.27 ± 0.688	427.054	0.00 (<0.05)
	Female	2.00 ± 0.00		
Age	18 <30	1.10 ± 1.656	119.366	0.00 (<0.05)
	30 <40	2.00 ± 0.00		
	40 <50	2.00 ± 0.00		
	50 <60	2.00 ± 0.00		
	60 <70	2.00 ± 0.00		
Area of Residence	Urban	10.00 ± 0.000	482.610	0.00 (<0.05)
	Rural	2.00 ± 0.594		
	Others	0.00 ± 0.000		
	Collage	0.67 ± 0.470		0.00 (<0.05)
Education	Graduate	1.64 ± 0.481	224.046	
	Postgraduates	2.00 ± 0.000		
	School	2.00 ± 0.000		
	Uneducated	2.00 ± 0.000		
Marital Status	Married	0.74 ± 0.438	41.899	0.00 (<0.05)
	Single	1.92 ± 0.274		
Employment Status	Full-Time	0.72 ± 0.449	231.776	0.00 (<0.05)
	Housewife	1.00 ± 0.000		
	Part-Time	1.86 ± 0.344		
	Retired	2.00 ± 0.000		
	Seeking-opportunities	2.00 ± 0.000		
	Student	1.82 ± 0.463		
	Unemployed	1.71 ± 0.681		
Native Language	Balochi	0.00 ± 0.000	360.161	0.00 (<0.05)
	Hindko	0.00 ± 0.000		
	Pashto	0.76 ± 0.430		
	Punjabi	1.48 ± 0.502		
	Saraiki	2.00 ± 0.000		
	Shina	2.00 ± 0.000		
	Sindhi	2.00 ± 0.000		
	Urdu	2.00 ± 0.681		

### Attitude of Participants

The participants' attitudes toward the preventive measures to limit the spread of COVID-19 and their responses are presented in Tables 6, 7, and Supplementary Table 1 and Supplementary 4. In general, the majority of participants had a good attitude toward the various preventive measures asked. About 95.8% of the participants were assured that Pakistan would take control over the pandemic of COVID-19 successfully. In comparison, the attitudes about the lockdown in the country were 87.3% positive, and 95.8% of participants favored a stringent policy if it helped to protect the general population from the devastating infection.

**Table 6:** Attitudes toward COVID-19 (*N* = 734).

**Questions**	**Responses**	**Frequency**	**Percent**	**Valid percent**	**Cumulative percent**
i) Do you have confidence that Pakistan can win the battle against the COVID-19 virus?	No	14	1.9	1.9	1.9
	Not sure	17	2.3	2.3	4.2
	Yes	703	95.8	95.8	100.0
ii) In no circumstance should there be curfews	No	61	8.3	8.3	8.3
	Not sure	32	4.4	4.4	12.7
	Yes	641	87.3	87.3	100.0
iii) I am in favor of a strict policy if it helps to protect the vulnerable	No	17	2.3	2.3	2.3
	Not sure	14	1.9	1.9	4.2
	Yes	703	95.8	95.8	100.0

**Table 7:** Association of attitude with demographic variables of respondents (*N* = 734).

**Variables**	**Categories**	**Attitude score (Mean+-SD)**	**t/F**	**p-value**
Gender	Male	2.74 ± 0.712	86.822	0.00 (<0.05)
	Female	3.00 ± 0.00		
Age	18 <30	2.68 ± 0.781	10.946	0.00 (<0.05)
	30 <40	3.00 ± 0.00		
	40 <50	3.00 ± 0.00		
	50 <60	3.00 ± 0.00		
	60 <70	3.00 ± 0.00		
Area of Residence	Urban	3.00 ± 0.000	299.071	0.00 (<0.05)
	Rural	2.74 ± 0.640		
	Others	0.00 ± 0.000		
	Collage	2.38 ± 0.994		0.00 (<0.05)
Education	Graduate	3.00 ± 0.000	47.787	
	Postgraduates	3.00 ± 0.000		
	School	3.00 ± 0.000		
	Uneducated	3.00 ± 0.000		
Marital Status	Married	2.51 ± 0.052	41.899	9.511 (<0.05)
	Single	3.00 ± 0.000		
Employment Status	Full-Time	2.47 ± 0.944	19.951	0.00 (<0.05)
	Housewife	3.00 ± 0.000		
	Part-Time	3.00 ± 0.000		
	Retired	3.00 ± 0.000		
	Seeking opportunities	3.00 ± 0.000		
	Student	3.00 ± 0.000		
	Unemployed	3.00 ± 0.000		
Native Language	Balochi	0.00 ± 0.000	52.562	0.00 (<0.05)
	Hindko	0.00 ± 0.000		
	Pashto	2.57 ± 0.834		
	Punjabi	3.00 ± 0.000		
	Saraiki	3.00 ± 0.000		
	Shina	3.00 ± 0.000		
	Sindhi	3.00 ± 0.000		
	Urdu	3.00 ± 0.000		

## Discussion

COVID-19 is a new, fast-evolving worldwide health concern that affects all areas of society. It is critical to persuade the general population to adopt precautionary measures, which are completely dependent on accurate information of the pandemic and appropriate general populations reaction. Numerous researches have been performed out to assess the level of KAP concerning infectious disease epidemics and pandemics including Severe acute respiratory syndrome (SARS), Influenza, Swine Flu, and Middle East respiratory syndrome (MERS). Recent studies on KAP toward COVID-19 among people in China (19), the United States (20), Tanzania (21), Kenya (22), Ethiopia (23), Saudi Arabia (24), and Nepal (25) have been reported.

Jilani et al. conducted a study to research the impact of information sharing on long-term performance in Bangladesh and concluded that knowledge concealment did not affect the impact of knowledge sharing on employees' ambidexterity (26). As a result, the purpose of this research was to explore the general population's knowledge, attitudes, practices, and anxiety about COVID-19 in Pakistan. This research also revealed several demographic factors linked to KAP's response to the COVID-19 pandemic. This research might give information to help healthcare officials and policymakers in establishing policies and awareness programs to limit the spread of the new COVID-19 pandemic.

Several web KAP studies have been done in Pakistan, with a focus on the educated population, including healthcare workers and university graduates. The results of previous studies found that participants were well-versed on common signs and symptoms (84–93%) and COVID-19 prevention measures (72–92%) (1). However, according to our findings, more than 90% of participants were aware of the common signs and symptoms Another community-based survey revealed relatively inadequate illness awareness, with just 45.3 %of the sample group viewing close physical contact as a risk factor for virus spread (27).

In our study, 95.5% of the respondents were hopeful that the fight against the COVID-19 pandemic would be successfully won and controlled in Pakistan through mitigation and prevention efforts. A significant proportion was revealed in China's recent survey, with 90.8% believing that COVID-19 will be successfully managed and 97.1% believing that China can win the battle against the SARS-CoV-2 (19). While according to another study in Pakistan, 67.3 % were confident that COVID-19 would be effectively managed; nevertheless, this was very low in comparison to our study (28).

In our study, the predominant portion (80%) were male participants which were similar to the studies conducted by other researchers (1), while some studies recruited more female participants (19, 21). The symptoms of the SARS-CoV-2 were investigated in this work, similar to the one conducted by others (1). In our study, 98.0% of participants reported that they avoided gathering in groups and eating or drinking in a public place throughout COVID-19, COVID-19, which was comparable to the research carried out by (1). We observed that 90.5% cleaned their hands regularly. Our findings are consistent with research done in Jordan, and Pakistan which found that frequent handwashing and mask-wearing are preventive measures used by the Jordanian and Pakistani population (29, 30). Another study from China found that participants had higher information, a positive attitude, and excellent health habits to prevent fatal infectious illnesses (31). In our study, the majority of participants (about 99.0%) used facemasks in public places and did not share personal items, which was higher than the findings of other studies (19, 25). In our study, a high proportion of the respondents (89%) thought that media coverage of this disease is exaggerated. A recent study in Karachi found that a large number of people (84 percent) think that false news about COVID-19, circulating on social media, is generating fear in the general population (32). These aspects of human life are governed by the trinity of general populations KAP, and the three components work together to form the complex system of life. As a result, they are all linked together in such a way that evidence of increased knowledge, changes in attitudes, and standard practices can all be traced back to pandemic impact prevention.

Concerns were expressed by more than half of the participants (52.5%), who were concerned about getting the disease; this ratio is a bit lower as compared to the Chinese population which is 54% in a recent report (33). There was a higher level of anxiety and susceptibility to getting and spreading the infection to oneself and family members in our study population. One possible explanation is that the general population did not fully comply with the government's preventive procedures, which resulted in the spread of COVID-19 fear.

The government of Pakistan responded well to the pandemic and succeeded in minimizing the losses. These preventive precautions may largely be ascribed to the government of Pakistan's stringent preventative and control measures, including banning of general populations gatherings and, a tight lockdown to stop the spread of COVID-19. The suspension of all incoming flights at airports, the closure of educational institutions, and the importation of 1,000 ventilators until the end of June 2020 are all significant steps (34). The success of the government of Pakistan in containing the contagion might also be the result of the people's thorough understanding of the COVID-19 virus's high virulence and speed of transmission. A tiny portion of the respondents (0.5%) reported that they do not wear face masks and 1.5% of participants gather in groups and do not avoid eating or drinking in a general population's place throughout COVID-19. Around 0.1% of participants mentioned that they do not wash their hands and stopped shaking their hands during the pandemic. These figures are a bit lower than the findings of the recent study (23). In research on KAP toward COVID-19, similar results were observed among the Nepalese population (25). Prevention is the best way to mitigate the effects of the COVID-19 pandemic, and general populations aware of the importance of SARS-CoV-2 preventive measures is critical, disseminating accurate information to the general populations about the situation of COVID-19 in Pakistan is critical for spreading awareness of the possible risks and the best code of behavior throughout COVID-19. Because of a lack of internet access and online health information, vulnerable populations in Pakistan affected by the COVID-19 epidemic, such as senior citizens and rural residents, are more likely to have low knowledge, negative attitudes, and ineffective prevention during the pandemic. As a result, in Pakistan today, KAP toward COVID-19 among the vulnerable population requires special attention. Furthermore, the study's sample was not representative. Another limitation was the lack of a detailed evaluation of KAP toward COVID-19, which could have been accomplished through focus group interviews and in-depth interviews for a more in-depth understanding.

### Implications

In the COVID-19 pandemic, the majority of research participants adopted social isolation measures, regular hand washing, and enhanced personal hygiene routines. Pakistani population showed a predicted degree of awareness of the COVID-19 and applied suitable anti-spread measures. Furthermore, specific training programs for government and local employees may enhance their knowledge of COVID-19 risks and preventative methods, allowing them to provide adequate care to their family members while also keeping themselves safe from the virus. Positively, nations like Pakistan may limit the spread of COVID-19 infection through developing efficient COVID-19 management and prevention programs at the government. To tackle the infection, WHO, CDC, local governments of Pakistan and other health organization plans should be implemented in this respect, everybody should work together to end the pandemic.

### Limitations

It was an online survey, and because it was shared on social media, there were probably numerous individuals in Pakistan who were unable to see or access it. We unintentionally overlooked the viewpoint of individuals who do not utilize the internet or social media. Moreover, the replies were based on honesty and were influenced through recall ability; hence, they may be subject to bias recall. We recruited participants via social media channels rather than a representative sample from the whole country, so our data may not be able to be generalized”.

### Conclusions

This study shows that participants have an overall good KAP toward COVID-19, some populations are less knowledgeable than others, with Pakistanis having varying opinions regarding the pandemic. Furthermore, given the recent increase in COVID-19 cases in Pakistan, there is a risk of compliance in procedures for preventative precautions. Following these guidelines and disseminating the right information requires complex awareness campaigns and educational interventions that focus on safe health practices and proper evidence-based information about this disease.

## Data Availability Statement

The original contributions presented in the study are included in the article/Supplementary Material, further inquiries can be directed to the corresponding authors.

## Author Contributions

All the authors contributed to the preparation of this manuscript. SK, MK, TU, JA, DW, MJ, KH, MAR, KS, and DW wrote the article, final editing, and preparation of the manuscript for submission. SK, DW, and XJ revised the manuscript. All authors read and approved the final article.

## Funding

This work was supported by the National Natural Science Foundation of China (Nos. 81802718 and U1504817), the Foundation of Science and Technology Department of Henan Province, China (Nos. 192102310151 and 202102310480), and the training Program for Young Backbone Teachers of Institution of Higher Learning in Henan Province, China (No. 2020GGJS038).

## Conflict of Interest

The authors declare that the research was conducted in the absence of any commercial or financial relationships that could be construed as a potential conflict of interest.

## Publisher's Note

All claims expressed in this article are solely those of the authors and do not necessarily represent those of their affiliated organizations, or those of the publisher, the editors and the reviewers. Any product that may be evaluated in this article, or claim that may be made by its manufacturer, is not guaranteed or endorsed by the publisher.
