# Supplementary material for: Assessment of General Populations Knowledge, Attitude, and Perceptions Toward the Coronavirus Disease (COVID-19): A Cross-Sectional Study From Pakistan
Source: Front Med (Lausanne). 2021 Dec 24;8:747819. doi: 10.3389/fmed.2021.747819 (PMC8754052; doi:10.3389/fmed.2021.747819)
Supplement: Supplementary file 1 [file Table_1.DOC]

|  | | Knowledge | | | | | | | | | | Attitude | | | | | | | | | | Perception | | | | | | | | | | |
| --- | --- | --- | --- | --- | --- | --- | --- | --- | --- | --- | --- | --- | --- | --- | --- | --- | --- | --- | --- | --- | --- | --- | --- | --- | --- | --- | --- | --- | --- | --- | --- | --- |
| Variables | Categories | B | P-Value | OR | | | 95% CI | | | B | | | P-Value | | OR | 95% CI | | | | | B | | | P-Value | | OR | | | 95% CI | | | |
|  |  |  |  |  |  |  | Lower Bound | Upper Bound | |  |  |  |  |  |  | Lower Bound | | Upper Bound | |  | | |  | |  | | | Lower Bound | | | Upper Bound | |
| Residence |  |  |  | | |  | | |  | |  | | |  | | |  | |  | |  | | | | | |  | | |  | |  |
|  | Rural | -21.481 | .998 | .000 | | | .000 | .000 | | -24.144 | | | .998 | | .000 | .000 | | . | | -22.146 | | | .998 | | .000 | | | .000 | | | . | |
|  | Urban | .000 | 1.000 | 1.000 | | |  |  | | .000 | | | 1.000 | | 1.000 | .000 | | . | | .000 | | | 1.0 | | 1.000 | | | .000 | | | . | |
|  | Single |  |  |  | | |  |  | |  | | |  | |  |  | |  | |  | | |  | |  | | |  | | |  | |
|  | Married |  |  |  | | |  |  | | -18.262 | | | .996 | | .000 | .000 | | . | |  | | |  | |  | | |  | | |  | |
|  |  |  |  |  |  |  |  |  |  |  |  |  |  |  |  |  |  |  |  |  |  |  |  |  |  |  |  |  |  |  |  |  |
| Education | Graduate | -20.925 | .998 | .000 | .000 | | | . | |  | | |  | |  |  | |  | | -20.259 | | | .998 | | .000 | | | .000 | | | . | |
|  | Postgraduate | .000 | 1.000 | 1.000 | .000 | | | . | |  | | |  | |  |  | |  | | .000 | | | 1.000 | | 1.000 | | | .000 | | | . | |
|  | school | .000 | 1.000 | 1.000 | .000 | | | . | |  | | |  | |  |  | |  | | .000 | | | 1.000 | | 1.000 | | | .000 | | | . | |
|  | Uneducated | .000 | 1.000 | 1.000 | .000 | | | . | |  | | |  | |  |  | |  | | .000 | | | 1000 | | 1.000 | | | .000 | | | . | |
