# Supplementary material for: Assessment of General Populations Knowledge, Attitude, and Perceptions Toward the Coronavirus Disease (COVID-19): A Cross-Sectional Study From Pakistan
Source: Front Med (Lausanne). 2021 Dec 24;8:747819. doi: 10.3389/fmed.2021.747819 (PMC8754052; doi:10.3389/fmed.2021.747819)
Supplement: Supplementary file 2 [file Table_2.DOC]

Table 2. Association between Demographic Variables and Knowledge


Variables	


Categories	               

             Knowledge	
		Poor Knowledge %age
(n)	Good Knowledge %age 
(n)	Chi-Square
Value	p-value	


Residence	Others	100% (16)	0% (0)	


163.562	

 0.00 
 
(<0.05)	
	Rural	24.1% (100)	75.9% (315)			
	Urban	0% (0)	100% (303)			
	Total 	15.8% (116)	84.2% (618)			
Gender	Male	19.5%(116)	80.5%(478)	

32.472	
0.00 
 
(<0.05)	
	Female	    0.0%  (0)	100.0%(140)			
	Total	15.8% (116)	84.2%(618)			
Age	18–< 30	24.3%(116)	
75.7%(362)	


73.787	


0.00 
 
(<0.05)	
	30–< 40	
0.0%(0)	
100.0%(165)			
	40–< 50	
0.0%(0)	
100.0%(59)			
	50–< 60	
0.0%(0)	
100.0%(17)			
	60–< 70	
0.0%(0)	
100.0%(15)			
	Total	
15.8%(116)	
84.2%(618)			


Education	College	46.8%(116)	53.2%(132)	


269.992	


0.00 
 
(<0.05)	
	Graduate	0.0%(0)	100.0%(279)			
	Postgraduate	0.0%(0)	100.0%(104)			
	School	0.0%(0)	100.0%(64)			
	Uneducated	0.0%(0)	100.0%(39)			
	Total	15.8%(116)	84.2%(618)			
Marital Status	Single	0.0%(0)	100.0%(419)	


183.261	


0.00 
 
(<0.05)	
	Married	36.8%(116)	63.2%(199)			
	Total	15.8%(116)	84.2%(618)			


Employment Status	Retired	0.0%(0)	100.0%(13)	


209.738	


0.00 
 
(<0.05)	
	Full-Time	39.9%(116)	60.1%(175)			
	Part-Time	0.0%(0)	100.0%(337)			
	Unemployed	0.0%(0)	100.0%(22)			
	Student	0.0%(0)	100.0%(17)			
	Housewife	0.0%(0)	100.0%(2)			
	Seeking Opportunities	0.0%(0)	100.0%(51)			
	Total	15.8%(116)	84.2%(618)			
Language	Balochi	100.0%(7)	0.0%(0)	


221.534	


0.00 
 
(<0.05)	
	Hindko	100.0%(2)	0.0%(0)			
	Pashto	36.3%(107)	63.7%(188)			
	Punjabi	0.0%(0)	100.0%(86)			
	Saraiki	0.0%(0)	100.0%(23)			
	Shina	0.0%(0)	100.0%(1)			
	Sindhi	0.0%(0)	100.0%(24)			
	Urdu	0.0%(0)	100.0%(296)			
	Total	15.8%(116)	84.2%(618)			

Table 3. Association between Demographic Variables and Attitude


Variables	


Categories	               

            Attitude	
		Poor Knowledge %age
(n)	Good Knowledge %age 
(n)	Chi-Square
Value	p-value	


Residence	Others	100% (16)	0% (0)	


376.580	

 0.00 
 
(<0.05)	
	Rural	3.6%% (15)	96.4% (400)			
	Urban	0% (0)	100% (303)			
	Total 	4.2% (31)	95.8% (703)			
Gender	Male	5.2% (31)	94.8% (563)	

7.629	
0.00 
 
(<0.05)	
	Female	    0.0%  (0)	100.0%(140)			
	Total	4.2% (31)	95.8%(703)			
Age	18–< 30	       6.5% (31)	
93.5% (447)	


17.335	


0.00 
 
(<0.05)	
	30–< 40	
0.0%(0)	
100.0%(165)			
	40–< 50	
0.0%(0)	
100.0%(59)			
	50–< 60	
0.0%(0)	
100.0%(17)			
	60–< 70	
0.0%(0)	
100.0%(15)			
	Total	
4.2% (31)	
95.8% (703)			


Education	College	12.5% (31)	87.5%(217)	


63.429	


0.00 
 
(<0.05)	
	Graduate	0.0%(0)	100.0%(279)			
	Postgraduate	0.0%(0)	100.0%(104)			
	School	0.0%(0)	100.0%(64)			
	Uneducated	0.0%(0)	100.0%(39)			
	Total	4.2% (31)	95.8% (703)			
Marital Status	Single	0.0%(0)	100.0%(419)	


43.053	


0.00 
 
(<0.05)	
	Married	9.8%(31)	90.2% (284)			
	Total	4.2% (31)	95.8%(703)			


Employment Status	Retired	0.0%(0)	100.0%(13)	


49.273	


0.00 
 
(<0.05)	
	Full-Time	10.7% (31)	89.3% (260)			
	Part-Time	0.0%(0)	100.0%(337)			
	Unemployed	0.0%(0)	100.0%(22)			
	Student	0.0%(0)	100.0%(17)			
	Housewife	0.0%(0)	100.0%(2)			
	Seeking Opportunities	0.0%(0)	100.0%(51)			
	Total	4.2% (31)	95.8% (703)			
Language	Balochi	100.0%(7)	0.0%(0)	


230.687	


0.00 
 
(<0.05)	
	Hindko	100.0%(2)	0.0%(0)			
	Pashto	7.5% (22)	92.5% (173)			
	Punjabi	0.0%(0)	100.0%(86)			
	Saraiki	0.0%(0)	100.0%(23)			
	Shina	0.0%(0)	100.0%(1)			
	Sindhi	0.0%(0)	100.0%(24)			
	Urdu	0.0%(0)	100.0%(296)			
	Total	4.2% (31)	95.8% (703)			

Table 4. Association between Demographic Variables and Perception


Variables	


Categories	               

            Perception	
		Poor Knowledge %age
(n)	Good Knowledge %age 
(n)	Chi-Square
Value	p-value	


Residence	Others	100% (16)	0% (0)	


175.623	

 0.00 
 
(<0.05)	
	Rural	15.7% (65)	84.3% (350)			
	Urban	0% (0)	100% (303)			
	Total 	11.0% (81)	89.0% (653)			
Gender	Male	13.6% (81)	86.4% (513)	

21.459a	
0.00 
 
(<0.05)	
	Female	    0.0%  (0)	100.0%(140)			
	Total	11.0% (81)	89.0%(653)			
Age	18–< 30	       16.9%(81)	
83.1% (397)	


48.762	


0.00 
 
(<0.05)	
	30–< 40	
0.0%(0)	
100.0%(165)			
	40–< 50	
0.0%(0)	
100.0%(59)			
	50–< 60	
0.0%(0)	
100.0%(17)			
	60–< 70	
0.0%(0)	
100.0%(15)			
	Total	
11.0% (81)	
89.0% (653)			


Education	College	32.7% (81)	67.3% (167)	


178.424	


0.00 
 
(<0.05)	
	Graduate	0.0%(0)	100.0%(279)			
	Postgraduate	0.0%(0)	100.0%(104)			
	School	0.0%(0)	100.0%(64)			
	Uneducated	0.0%(0)	100.0%(39)			
	Total	11.0% (81)	89.0% (653)			
Marital Status	Single	0.0%(0)	100.0%(419)	


121.108	


0.00 
 
(<0.05)	
	Married	25.7% (81)	74.3% (234)			
	Total	11.0% (81)	89.0% (653)			


Employment Status	Retired	0.0%(0)	100.0%(13)	


138.605	


0.00 
 
(<0.05)	
	Full-Time	27.8% (81)	72.2% (210)			
	Part-Time	0.0%(0)	100.0%(337)			
	Unemployed	0.0%(0)	100.0%(22)			
	Student	0.0%(0)	100.0%(17)			
	Housewife	0.0%(0)	100.0%(2)			
	Seeking Opportunities	0.0%(0)	100.0%(51)			
	Total	11.0% (81)	89.0% (653)			
Language	Balochi	100.0%(7)	0.0%(0)	


179.618	


0.00 
 
(<0.05)	
	Hindko	100.0%(2)	0.0%(0)			
	Pashto	24.4% (72)	75.6% (223)			
	Punjabi	0.0%(0)	100.0%(86)			
	Saraiki	0.0%(0)	100.0%(23)			
	Shina	0.0%(0)	100.0%(1)			
	Sindhi	0.0%(0)	100.0%(24)			
	Urdu	0.0%(0)	100.0%(296)			
	Total	11.0% (81)	89.0% (653)			
